# Human Cytochrome P450 1, 2, 3 Families as Pharmacogenes with Emphases on Their Antimalarial and Antituberculosis Drugs and Prevalent African Alleles

**DOI:** 10.3390/ijms24043383

**Published:** 2023-02-08

**Authors:** Chiratidzo R. Chamboko, Wayde Veldman, Rolland Bantar Tata, Birgit Schoeberl, Özlem Tastan Bishop

**Affiliations:** 1 Research Unit in Bioinformatics (RUBi), Department of Biochemistry and Microbiology, Rhodes University, Makhanda 6139, South Africa; 2 Translational Medicine, Novartis Institutes for BioMedical Research, 220 Massachusetts Ave, Cambridge, MA 02139, USA

**Keywords:** CYP metabolism, polymorphisms, SNPs, missense mutations, personalized medicine

## Abstract

Precision medicine gives individuals tailored medical treatment, with the genotype determining the therapeutic strategy, the appropriate dosage, and the likelihood of benefit or toxicity. Cytochrome P450 (CYP) enzyme families 1, 2, and 3 play a pivotal role in eliminating most drugs. Factors that affect CYP function and expression have a major impact on treatment outcomes. Therefore, polymorphisms of these enzymes result in alleles with diverse enzymatic activity and drug metabolism phenotypes. Africa has the highest CYP genetic diversity and also the highest burden of malaria and tuberculosis, and this review presents current general information on CYP enzymes together with variation data concerning antimalarial and antituberculosis drugs, while focusing on the first three CYP families. Afrocentric alleles such as CYP2A6*17, CYP2A6*23, CYP2A6*25, CYP2A6*28, CYP2B6*6, CYP2B6*18, CYP2C8*2, CYP2C9*5, CYP2C9*8, CYP2C9*9, CYP2C19*9, CYP2C19*13, CYP2C19*15, CYP2D6*2, CYP2D6*17, CYP2D6*29, and CYP3A4*15 are implicated in diverse metabolic phenotypes of different antimalarials such as artesunate, mefloquine, quinine, primaquine, and chloroquine. Moreover, CYP3A4, CYP1A1, CYP2C8, CYP2C18, CYP2C19, CYP2J2, and CYP1B1 are implicated in the metabolism of some second-line antituberculosis drugs such as bedaquiline and linezolid. Drug–drug interactions, induction/inhibition, and enzyme polymorphisms that influence the metabolism of antituberculosis, antimalarial, and other drugs, are explored. Moreover, a mapping of Afrocentric missense mutations to CYP structures and a documentation of their known effects provided structural insights, as understanding the mechanism of action of these enzymes and how the different alleles influence enzyme function is invaluable to the advancement of precision medicine.

## 1. Introduction

Precision medicine has been of interest to many scientists over recent years who envision a world where each person or groups of people can receive the right medications for their medical condition based on their genotype. Ideally, the genotype determines the therapeutic strategy, the appropriate dosage and the likelihood of benefit or toxicity. Additional factors affect patient response to medications such as age, sex, disease stage, and environment. However, genetic makeup, [1,2,3] is a critical factor, as it directly determines the likelihood to respond to a targeted therapy or how drugs are metabolized [2,4,5]. Pharmacogenomics, therefore, forms an integral part of precision medicine since it combines both pharmacology and genomics to determine how differences in the human genome, especially the ones located at pharmacogenes, affect drug metabolism and efficacy [5,6,7]. Such genomic differences are caused by genetic variations due to mutations such as single nucleotide polymorphisms (SNPs), insertions and deletions, or copy number variations [8]. These polymorphisms may be population specific or selected for across different populations, leading to slight differences in the expressed proteins or enzymes responsible for drug metabolism that are encoded by pharmacogenes [5,9,10]. The resultant effects of these differences on enzyme activity may be visible as minimal to no therapeutic response in some individuals while others may suffer from adverse drug reactions [7,11]. To minimize harmful side effects, while maximizing the therapeutic effectiveness of drugs in each patient, it is important to understand the effects of genetic variations on pharmacogenes and their associations with variable drug responses in different populations [12]. For instance, identifying the proto-oncoprotein *KRAS* genotype of a patient provides information about disease aggressiveness, which drugs to be prescribed (e.g., KRAS-G12C inhibitors sotorasib and adagrasib), and drug sensitivity [13].

Functional characterization of pharmacogenes segregates them into three main classes: drug transporters (e.g., P-glycoprotein 1 (P-gp), ATP binding cassette subfamily B member 1 (ABCB1)); drug metabolizers (cytochrome P450 enzymes (CYPs)); and drug targets (e.g., human epidermal growth factor receptor 2 (HER2)). There exists an intricate interplay between these classes of pharmacogenes and genetic variations involving any of them can potentially influence treatment outcomes or pharmacogenetic phenotypes [14,15,16].

The main drug metabolizing enzymes in humans belong to the CYP enzyme superfamily, and are actively involved in phase 1 metabolism of most therapeutic drugs and xenobiotics [17]. CYP enzymes are highly polymorphic, with newly discovered alleles being updated regularly in the Pharmacogene Variation (PharmVar) Consortium database [18]. Certain SNPs from CYP families have been shown to have a great impact on CYP function [19,20], while some have exerted no noticeable change in enzyme activity [1].

In this review, we first provide a general overview of CYP enzymes, and then focus on the three human CYP families (CYP1, 2, and 3) that are particularly important in the metabolism of drugs. We detail the enzymes of these three families, compare similarities and differences between them and combine the data with the 3D structural information where applicable. We further provide up to date variation data attached to these proteins. Although the diversity of CYP enzymes within the African Continent is well appreciated, with implications on drug resistance and adverse drug reactions [20,21], little is known about the underlying mechanisms responsible for such observations. Hence, as a next step, we outline the available data from the African perspective. Firstly, we convene on CYPs that are particularly involved in the metabolism of drugs used in the treatment of two important infectious diseases; malaria and tuberculosis, for which Africa shares the greatest burden [21,22]. Secondly, we review Afrocentric polymorphisms of CYP enzymes with specific focus on potential or known effects on antimalarial and antituberculosis drugs; and discuss the connections between the variations and their positions with possible explanations to their structural/functional effects. Collectively, in this review, by gathering the existing information and by pointing out the current gaps from different aspects, we aim to create a baseline understanding of CYP-drug-variation with specific emphases on malarial or tuberculosis drugs as well as the alleles that are prevalent in the African Continent. In the long term, we believe information shared here could contribute to the development of suitable therapeutic drugs and drug combinations for the treatment of prevailing infectious diseases within the Continent and elsewhere.

## 2. CYP Enzymes

CYP enzymes are a superfamily of heme-thiolate proteins that are present in all kingdoms of life [23]. They are essential for the production of steroids, cholesterol, prostacyclins, and thromboxane A2 [1,17], and partake in the detoxification of foreign chemicals and the metabolism of drugs [17,24,25]. In humans, they are mainly found within the mitochondria and endoplasmic reticulum of liver cells; however, they can be located in many other parts of the body including the kidneys, small intestine, placenta, and lungs [1,17]. There are 57 known human genes that encode for the different CYP enzymes, and these have been classified into 18 protein families [26]. The enzymes in the first three families (CYP1, CYP2, and CYP3) are responsible for the metabolism of 70–80% of clinical drugs [17,24] and most foreign chemicals; however, within these families, only enzymes CYP1A1, CYP1A2, CYP2A6, CYP2B6, CYP2C8, CYP2C9, CYP2C18, CYP2C19, CYP2D6, CYP2E1, CYP3A4, and CYP3A5 notably metabolize most of clinical drugs [1,27,28]. Most CYPs metabolize more than one drug, and also, a drug is generally metabolized by more than one CYP enzyme [19].

### 2.1. Human CYP Enzyme Nomenclature

The establishment of standardized CYP enzyme nomenclature dates back to 1987 [29]. At the time, enzymes which belonged to the P450 gene superfamily were classified based on amino acid sequence similarities such that enzymes from different gene families shared ≤36% similarity while those from the same subfamily had ≥70% similarity [29,30]. As more data became available, the classification and nomenclature of CYP enzymes also evolved [29,30,31]. While the original nomenclature was built on only 65 characterized P450 genes [29], the final nomenclature is based on 221 P450 genes [32]. The current classification of CYP enzymes relies more on phylogenetic tree clustering rather than the arbitrary sequence identity values which were subject to changes based on data availability [30,32,33].

The current nomenclature is as follows: CYPxyz, where CYP = Cytochrome P450 and x = family (Arabic numeral), y = subfamily (Capital letter, if more than one exists), z = gene name (Arabic numeral), and this is followed by the letter *P* if the gene in question is a pseudogene [30,32].

After the CYP nomenclature was agreed upon to gene level, allele representation remained ambiguous until later when the currently acceptable method was proposed for CYP2D6 [32,34]. Here, the name is maintained to the gene level followed by an asterisk, then the allele in Arabic numerals which may be followed by a capital letter denoting silent mutations in the gene as follows: CYPxyz*ij, where CYPxyz is as defined above, i = allele (Arabic numeral), j = silent mutation (Capital letter) [34,35,36]. Further updates have been applied to this method, particularly representing the silent alleles as three decimal point digits after the allele name as seen in the Pharmacogene Variation (PharmVar) Consortium database [18]. The consensus/reference allele, usually representing the major proportion of the population, confers normal activity of the CYP enzyme [35]. An allele is classified as a pharmacogenetic polymorphism if it occurs at a frequency of at least 1% in a population [19]. Sub-alleles are designated when an already-characterized sequence variant is discovered with additional non-causative variant/s [35]. These sub-alleles are given letters in addition to the number (e.g., CYP2B6*4A, CYP2B6*4B, CYP2B6*4C). However, when more than one effective variant exists on the same allele, the given allelic number is based on the variant causing the most serious effect, such as a splice defect (e.g., CYP2C19*2A). Unique allele numbers (e.g., CYP2B6*6) are given to combinations of variants that also occur on their own and are judged to be uniformly effective (e.g., different amino acid substitutions). Importantly, for practical purposes, the earliest alleles ever reported are not based on this nomenclature system as they have not been re-named.

### 2.2. Human CYP Enzyme Sequence, Structure, and Mechanism of Action

To understand ligand binding/selection and the general structure–function relationship of human CYP enzymes, one must have knowledge of the sequential, structural, and mechanistic features of CYPs.

Human CYP enzymes within families 1, 2, and 3 are known to have similar structure and function. Enzyme sequences within these families contain about 400–500 amino acids [28,37], and those that share ~40% sequence identity are grouped in the same family, while those sharing over 55% sequence homology are subgrouped together into the same sub-family [28,38]. According to [39] there are three conserved short regions/sequence motifs throughout the CYP superfamily. The first conserved region is the (A/G)XXXT which is located in helix I [37,39]. This is the oxygen binding and activation motif that contains the highly conserved threonine which plays an important role in the third step of the catalytic cycle of CYP enzymes [39]. The second most conserved region in CYP enzymes is the EXXR motif, which contains conserved glutamic acid and arginine that build a set of salt-bridge interactions which form the final tertiary structure of the enzymes [39]. The FXXGXXXCXG region is the third most conserved one in the CYP enzyme superfamily [40]. It is a heme-binding domain that contains phenylalanine, glycine, and cysteine, the three most conserved residues in the CYP superfamily, of which the conserved cysteine plays the role of the axial ligand to the heme [39,40].

In contrast to prokaryotic CYPs, eukaryotic CYPs are membrane proteins. Specifically, they are bitopic membrane proteins (spans the lipid bilayer only once) that are found on the cytoplasmic side of the endoplasmic reticulum or on the matrix side of the inner mitochondrial membrane [41]. They have an N-terminal transmembrane helical anchor that extends across the membrane bilayer and is joined by a flexible linker to a large globular domain that partly sits inside the membrane [42]. CYPs cannot function on their own and must bind with a redox partner for electron transfer [43]. These complexes are difficult to crystallize, resulting in limited protein–protein redox complexes in the Protein Data Bank (PDB). During CYP electron transport, reducing equivalents from nicotinamide adenine dinucleotide phosphate (NADPH) are transferred to molecular oxygen [44,45].

CYPs are typically divided into two major classes, class I and class II, based on their cellular location and their redox partners. Class l encompasses mitochondrial and bacterial CYPs which utilize two different redox partners: an iron–sulfur protein (ferredoxin/adrenodoxin) and a flavin-containing reductase (ferredoxin/adrenodoxin reductase). On the other hand, class II CYPs are microsomal monooxygenases that accept electrons from NADPH-cytochrome P450 oxidoreductase (POR). POR is a single polypeptide consisting of one molecule of flavin adenine dinucleotide (FAD) and one molecule of flavin mononucleotide (FMN) [43]. POR transfers electrons from the two-electron donor NADPH to the CYP heme group, with the FAD functioning as a dehydrogenase flavin and FMN as an electron carrier. The CYP enzymes reviewed in here belong to class II.

**Figure 1:**
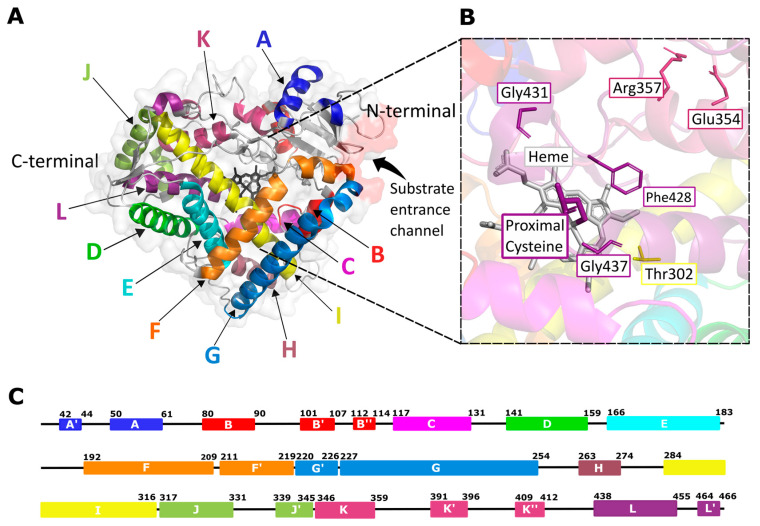
(**A**) General structure of CYP enzymes. CYP2C8 (PDB ID: 2NNJ) is used to map the 12 major helices denoted A-L in different colors. Substrate entrance site is highlighted in red and an arrow pointing towards the entrance is in black. (**B**) Conserved catalytically important residues mapped onto the CYP structure, and the cysteine that acts as a proximal axial thiolate ligand for the heme iron is highlighted. Threonine in the conserved motif (A/G)XXT is colored in yellow, while glutamic acid and arginine in the EXXR motif are colored in warm pink. Phenylalanine, glycine and cysteine in the conserved motif FXXGXXXCXG are all colored in purple. (**C**) CYP2C8 enzyme residue numbers are denoted, as an example, for the helices observed in CYP enzymes.

The fold structure of CYP enzymes is largely conserved throughout the superfamily [37,46]. All known 3D structures of these proteins have a general shape that includes 14 helices and loops denoted A–L (A, B, Β′, C, D, E, F, G, H, I, J, K, K′, L) [23] (Figure 1A). Other helices such as A′, B″, F′, G′, J′, K″, and L′ have been observed to occur in the CYP structure at times [46]. CYPs also contain 4 β-sheets—β1 (5 strands), β2 (2 strands), β3 (3 strands), β4 (2 strands) in their structures, with two additional sheets, β5 and β6, occurring sometimes [23,46]. Although the overall CYP structure is conserved, the size and shape of the active site can differ [43]. The B–C and F–G helix regions are the least conserved and contribute to substrate specificity, especially the B′ helix [43]. These regions change conformations for ligand entry, and together with the F–G loop, form a ‘‘roof” for the active site opposite to the heme ‘‘floor” [47,48]. The highest structural conservation in CYPs is centered around the heme–thiolate oxygen activation chemistry [43] (Figure 1B).

The CYP active site is situated in the protein hydrophobic core which is linked to the enzyme surface by channels [49,50] that substrates and O_2_ molecules enter, products exit, and water molecules move [51]. CYP enzymes contain a prosthetic heme group in their active site that is crucial for enzyme function during metabolism [43]. It is located between helices I and L and is covalently linked to the enzyme through the sulfur atom of a conserved cysteine residue that acts as the proximal axial thiolate ligand for the heme iron [43,52,53] (Figure 1B). The heme is regarded as a reactive center to activate oxygen and to oxidize the substrate [52]. Coordinated water gates controlled by the propionate side chains of heme, and their salt bridge partners facilitate water movement [43]. A commonly conserved acid/alcohol pair that is important for the P450 active cycle is situated on the I-helix [54], whilst the loop before the L-helix houses the cysteine axial thiolate ligand [52]. The oxidative prowess of CYP enzymes is linked to the formation of a coupled high-valent iron (IV)–oxo porphyrin π-cation radical species (Compound I) involving the iron ion of the enzyme’s heme group, dioxygen, two reducing, and two proton equivalents supplied within the CYP enzyme’s catalytic cycle [51,55,56]. The highly conserved β-bulge region (FXXGXXXCXG) containing the cysteine axial thiolate ligand is rigid so as to maintain a hydrogen-bonding distance from two peptide NH groups (seen in all CYPs) [43], even though the hydrogen-bonding geometry supports only one hydrogen-bond. These hydrogen-bonds play a role in controlling the heme iron redox potential [57,58], which would be too low for reduction by redox partners without the hydrogen-bonds. It is thought that in order to sustain a physiologically accessible range for the redox potential, the protein must have a satisfactory electrostatic environment around the cysteine ligand. While there is evidence pointing to Compound I as the ultimate oxidant in CYP enzymes, the oxidative versatility of the enzymes is likely linked to their plasticity, especially within the active site, and the ability of the catalytic cycle to be initiated by the entry of an oxidisable substrate into the active site [51]. On the other hand, unique features within the active sites/entry channels of different CYP enzymes—such as those due to mutations—modulate the stereo- and regioselectivity associated with their oxidation of different substrates and may result in different oxidized products of the same substrate [51,59,60].

**Figure 2:**
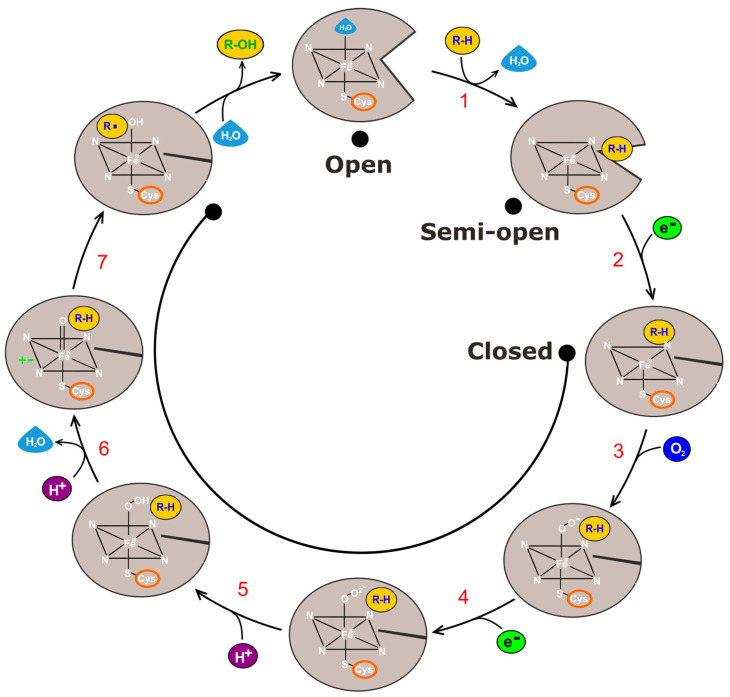
Mechanism of CYP enzymes in a seven-step catalytic cycle. Steps, explained in the text, are indicated with red numbers. Enzyme open, semi-open, and closed conformations at each stage denoted by a grey shape. The substrate, hydrogens, electrons, and oxygen are denoted by yellow, purple, green, and dark blue circles. Water is shown by a blue drop [51,78,79,83,84].

All CYP enzymes in the 18 human families identified are involved in phase 1 metabolism [61,62], where they metabolize various endogenous and exogenous compounds such as xenobiotics and environmental chemicals [63]. These enzymes function as monooxygenases [64,65], carrying out the metabolism of drugs through oxidation reactions, including hydroxylation, epoxidation, sulfoxidation, C–C bond cleavage, and desaturation reactions [66,67,68,69,70,71,72,73,74]. The overall CYP canonical reaction comprises the reductive scission of the O–O bond of atmospheric dioxygen to release a single molecule of water with the transfer of a single oxygen atom to the substrate [75,76]. The convoluted catalytic cycle has multiple reactions with transient intermediates, where the product of one reaction is the substrate for the next reaction by the same enzyme. During the mechanism, the heme iron changes spin states [77] due to the binding of substrate, as the outer orbital has one or more unpaired electrons giving it a higher energy state. The following is a short description of the typical seven-step catalytic cycle seen in CYP enzymes (Figure 2) [51,78,79]:

Before the substrate enters the enzyme, the heme iron is coordinated to a water molecule and is in a low-spin ferric resting state (S = 1/2). Step 1: The cycle starts when the substrate first enters the active site and interacts with the resting state. The water molecules leave the pocket [71] which detaches the aqua ligand resulting in the high-spin Fe^III^–heme complex (S = 5/2). Step 2: The now more positive redox potential [80,81] allows electron transfer from the reducing partner, to the now ferrous Fe^II^ complex. Step 3: As the ferrous Fe^II^ complex is a good O_2_ binder, it takes up an O_2_ molecule and is transformed into the oxyferrous complex. Step 4: As the oxyferrous complex is a good electron acceptor, it is reduced and is transformed into the peroxo complex. Step 5: Around this time, the water molecules that departed the active site pocket return through a water gate and create a water channel that protonates the peroxo complex to give Compound 0 (Cpd 0) [51], which is debated to be a putative oxidant [82]. Step 6: The negatively charged Cpd 0 is a good Lewis base that accepts an extra proton, freeing a water molecule, and forms Compound I (Cpd I) which is a ferryl (Fe^IV^)-oxo-π porphyrin cation radical and the ultimate oxidant. Step 7: Cpd I is thought to be responsible for the bond activation in the substrate via hydrogen abstraction, leading to substrate oxidation [73]. The product now leaves the pocket and a water molecule takes its place. The initial CYP enzyme resting/ferric state is now re-established and set for another cycle.

Dubey and Shaik 2019 [51] researched the determining factors that allow the sequence of steps to occur, using molecular dynamics (MD) and quantum-mechanical/molecular-mechanical (QM/MM) calculations on mostly bacterial CYPs, namely bacterial CYP_BM3_ and CYP_CAM_, but also human CYP3A4. Substrate binding, O_2_ entrance, reductase attachment, and gating were researched using MD simulations [85,86,87,88]. Water channel formation and reorganization generally need about 100 ns of simulation time. Substrate binding and protein–protein interactions need between 350–400 ns and 1000–1500 ns, respectively. MD snapshots are used for their QM/MM calculations [73,74], which calculate the active species and their chemical events and provide geometries, electronic structures, mechanisms, and energies of the species in their native protein environment. It was discovered that the CYP catalytic cycle is initiated by substrate binding and from then on each step is automatically coordinated due to entropy rise [71] and switchable weak interactions [51].

### 2.3. Existing Structural Information in the Protein Data Bank (PDB)

As of November 2022, Protein Data Bank (PDB) [89] has a total of 177 structures for CYP 1, 2, and 3 family members. The enzymes CYP1A2, CYP2D6, and CYP3A4 were among the first few enzymes within the CYP family to have structures in the database, with the first structure being deposited in 2003. More than a decade later, many of the 23 human CYP enzymes from families 1, 2, and 3 have structures available. Currently, the enzymes with no structures within the database are CYP2A7, CYP2C18, CYP2F1, CYP2J2, CYP2S1, CYP2U1, CYP2W1, and CYP3A43. The enzyme CYP3A4 has the largest structural data available, with 87 structures in PDB. CYP2A6 and CYP2D6 have the second largest number of structures available, with 14 structures each. The PDB also contains variants of CYP enzymes within these three families such as CYP2C9 alleles *2, *3, *8, and *30. A summary of the best structures for each enzyme in the three families is shown in Table S1.

The structures of CYP enzymes in PDB are either in an open, partially open or closed conformation [83]. The open conformation is usually associated with ligand free proteins and the closed conformations are proteins with a ligand bound; however, closed ligand-free structures and partially open ligand-bound structures have been determined [83,84]. Variations in the pre-helix A, the B–C and F–G regions have been defined to cause the different conformations of human CYP enzymes [83,84,90,91,92]. CYP2D6 in an open and closed conformation as an example, is shown in Figure S1. When closed, the pre-helix A G42 residue is 14.3 Å from helix F’ residue E222, but in an open conformation, the distance between helix F’ E222 residue and the pre-helix A residue G42 increases by 0.6 Å to 14.9 Å, thus opening the substrate channel for ease of access to the active site [84]. Subsequent changes in the adjacent regions including the F and G helices, and the helix B-C loop that contains helix B’ follow, creating a CYP2D6 open conformation structure [84].

#### Antimalarial and Antituberculosis Drugs Complexed with CYP Enzymes

Currently, 3D structural data of CYPs with antimalarial drugs are extremely scarce and non-existent for the antituberculosis drugs. Furthermore, CYP structural information with its functional proteins and membrane partners are not available. This information deficiency hinders the attempts to understand the changes in the drug metabolism due to specific variations.

Of the 177 structures available for human CYP enzymes in families 1, 2, and 3, only two structures are complexed with antimalarial drugs: CYP2D6 with quinine (4WNV) and CYP2D6 with Quinidine (4WNU). X-ray diffraction method was used to obtain the complexes, and they were refined to resolutions of 2.26 Å and 2.35 Å for the quinidine and quinine complexes, respectively [84]. These antimalarial drugs are inhibitors of CYP2D6 and are not naturally metabolized by this enzyme [84,93,94]. They are bound to the active site of CYP2D6, and several differences in binding conformations were discovered [84].

**Figure 3:**
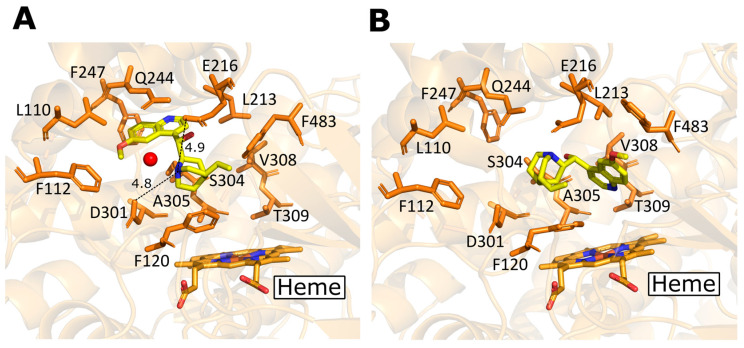
(**A**) CYP2D6 complexed with quinidine (PDB ID: 4WNU). (**B**) CYP2D6 complexed with quinine (PDB ID: 4WNV). Enzyme carbons are colored orange and ligand carbons are colored yellow. Oxygen, nitrogen, sulfur, iron, and nickel atoms are colored red, blue, yellow, orange, and green, respectively. Water molecule shown as a red sphere. Distances between the nitrogen in the quinuclidine ring of the bound quinidine with Asp-301 and Glu-21 are shown by dashed lines and distances are given in Å [84].

Quinine is a the diastereomer of quinidine, and it is known that these two compounds differ in configurations of the hydroxylated chiral carbon which connects the aromatic quinolone moieties and the protonated and positively charged bicyclic quinuclidine rings of both compounds [84]. As seen in Figure 3, due to the configurational differences between quinine and quinidine, the quinolone rings of the two compounds bind to significantly different positions within the CYP2D6 active site, which leads to different poses overall for the two compounds. Additionally, in the quinuclidine ring of the bound quinidine, the protonated nitrogen is bound to a water molecule in the entrance channel and is positioned almost equidistant between the negatively charged Glu-216 (4.9 Å) and Asp-301 (4.8 Å) side chains [84]. The differences observed for the binding of quinidine (Figure 3A) and quinine (Figure 3B) within CYP2D6, are contributing to the differences in inhibition of the two compounds [84]. It has been reported that while quinine is a potent inhibitor, quinidine is more potent [95]. More information on the complexes can be found in the article by Wang et al. 2015 [84].

### 2.4. Structural Differences in Human CYP 1, 2, 3 Enzymes

As indicated in the previous sections, the 3D structures of mammalian CYPs are generally conserved; however, some sequence and structural variations in the B–C helix, F–G helix, and L helix regions are known [96]. The differing shapes and sizes of the active site cavities of human CYPs are a result of discrepancies of the F–G and B’ helices. The dissimilarity of these regions causes some variability of enzyme function.

CYP family 1 enzymes generally metabolize polynuclear aromatic hydrocarbons, and the structures of human CYP1A1 [92], 1A2 [96], and 1B1 [96] possess narrow active site pockets that suit the size and planarity of polynuclear aromatic hydrocarbons. For example, CYP1A2 has a preference for small planar aromatic or heterocyclic amine ligands, engendered by active site-facing polar residues Thr118, Ser122, and Thr124 that are located in the B’–C helices region, as well as residues Thr223 (F helix) and Asp320 (I helix) that are found on the roof of the active cavity [96]. On the other hand, because CYP2A6 has a smaller active site pocket, it prefers to bind small aromatic ligands [97] with the help of the substrate-orienting residue Asn297 [98]. CYP2C9 prefers weakly acidic substrates [99] due to its disordered arrangement of the F–G loop region and an additional turn at the N-terminal side of helix A [100]. CYP2C9 has a smaller active site cavity than CYP2C8 but bigger than CYP2A6 [101]. With a comparatively small active site pocket, CYP2E1 prefers neutral compounds with low molecular weight and fatty acids [102]. In the CYP2E1 active site cavity, a conserved Asp295 is critical for substrate recognition and ligand binding. The very flexible CYP3A4 enzyme has a large binding cavity, allowing many structurally diverse ligands to bind [103]. This could be aided by the fact that the heme surface of CYP3A4 enzymes is much more exposed to substrates [47,104]. CYP3A4 has comparatively short F and G helices, and a cluster of large hydrophobic phenylalanine residues situated on the roof of the active site in the ligand-free structure that are distorted upon ligand binding [104,105,106]. The shorter CYP3A4 F-helix does not cross above the active site, meaning the active site can expand and contract due to the positional fluctuations of helices F′ and G′ as well as the flexibility of the long connector between helix F and F′ [105]. A high sequence identity between CYP3A4 and CYP3A5 result in similar structure; however, the CYP3A5 active site cavity has extra space between the helix F–F’ and helix G’–G connectors due to the shorter Leu108 in CYP3A5 (Phe108 in CYP3A4), as well as the longer CYP3A5 Phe210 (Leu210 in CYP3A4) which increases the size of the upper region of the substrate binding cavity [107].

## 3. CYP 1, 2, 3 Enzyme Families and Antimalarial Drugs

Antimalarial drugs are used either for chemoprevention or for the treatment of uncomplicated and severe malaria. Current indications include, sulfadoxine pyrimethamine (SP) for intermittent preventive treatment in pregnancy (IPTp) and infants (IPTi), and seasonal malaria chemoprevention (SMC) in children, with the latter including the addition of amodiaquine, atovaquone + proguanil are also indicated as prophylaxis for travelers to endemic areas [108]. Treatment of uncomplicated malaria is by use of artemisinine-based combination therapies (ACT) including artemether + lumefantrine, artesunate + either amodiaquine, mefloquine, SP, or pyronaridine, and dihydroartemisinin + piperaquine while severe malaria requires prior intravenous or intramuscular artesunate administration followed by oral ACT [108]. Finally, primaquine is recommended alongside ACT to reduce transmission while quinine and clindamycin are recommended for the treatment of uncomplicated malaria in pregnancy [108]. Diverse toxicity profiles are associated with some antimalarial drugs, for example quinine: rare cardiovascular toxicity and hypoglycemia, mefloquine: dose-related serious neuropsychiatric toxicity, pyrimethamine + dapsone: agranulocytosis, SP + amodiaquine: potentially fatal reactions, chloroquine + proguanil: mouth ulcers and gastrointestinal upset, halofantrine: cardiotoxicity [109,110].

Antimalarial drug metabolism may result in bioactivation, altered toxicity, or preparation of the drug for excretion [111]. Like with other xenobiotics, phase I metabolism of most antimalarial drugs is performed by CYP enzymes—particularly those belonging to the CYP 1, 2 and 3 families [1,112,113,114,115].

CYP enzymes function by catalyzing the oxidation of organic substrates, rendering the substrates more hydrophilic [51,55]. Following oxidation, the substrate either becomes bio-activated—like with the prodrugs amodiaquine and proguanil [111,116], directly excreted, or conjugated in phase II metabolism prior to excretion like with dapsone and artemisinins [28,117,118]. CYP metabolism may also result in more toxic oxidized products as seen with dapsone metabolism and hemolytic anemia and methemoglobinemia in humans [119]. Some of the antimalarial drugs on the market with known CYP enzymes as metabolizers and their oxidized products are presented in Table 1.

## 4. CYP 1, 2, 3 Enzyme Families and Antituberculosis Drugs

Months-long tuberculosis (TB) treatment involves the combination of several antituberculosis drugs to prevent the development of a resistant strain [2,140]. For drug-susceptible TB, international guidelines have advised the combination of all first-line drugs isoniazid, rifampicin, ethambutol, and pyrazinamide for 2 months followed by 4 months of isoniazid and rifampicin [141]. However, resistant strains of TB have emerged leading to the development and usage of second line drugs [142,143]. For multi-drug resistant (MDR) TB, a 6-month “BPaLM” regimen of bedaquiline, pretomanid, linezolid, and moxifloxacin may be used in patients older than 14 years of age with MDR/rifampicin-resistant (RR) TB who have not had previous exposure to bedaquiline, pretomanid, and linezolid [144]. Moxifloxacin can be omitted here (“BPaL”) if the TB is resistant to fluoroquinolones. A new drug in the same class as linezolid that is currently in testing, namely sutezolid, could have better therapeutic value than linezolid [145]. Unfortunately, with TB, drug associated toxicity is common due to the amount of antibiotics used and the long treatment period. Typically, second-line TB drugs are less effective and more toxic than the first-line drugs.

Drug-susceptible TB treatment has a 95% cure rate (in optimal conditions); however, several unclear concerns exist such as the high variability of response, the likelihood of drug underexposure, the high prevalence of drug-related toxicity, and the selection of multidrug-resistant strains [141]. There is a scarcity of early biomarkers for predicting treatment efficacy, cure, and the determination of patients needing prolonged treatment [146]. Inter-individual variability in the pharmacokinetics of anti-tubercular drugs may be responsible for the variability of response as it’s been shown to have major influence over the sterilizing effect and selection of phenotypic resistance. Low maximum plasma concentrations are linked to treatment failure, relapse, and acquired drug resistance, whereas high plasma concentrations are linked to hepatoxicity [147,148]. Therefore individual/personalized treatment based on patient pharmacogenetics is extremely important.

The frequency of hepatotoxicity in patients receiving anti-tubercular treatment is between 2% and 28% [149]. Toxic metabolites play a role in hepatotoxicity, but specific mechanism is not known. The human liver metabolizes most drugs, requiring several reactions and drug metabolizing enzymes [150]. The rate-limiting step for the clearance of drugs from the body are phase I metabolic reactions, then during phase II metabolic reactions the drug and its metabolites associate with endogenous substances and are released from the body. This action does have a detoxification effect; however, several active metabolites that are simultaneously produced can cause liver damage.

First-line antituberculosis drugs undergo different pathways during metabolism, but none have been discovered to be directly metabolized by any enzymes from the CYP families. However, metabolism of the first-line antituberculosis drug isoniazid is indirectly influenced by CYP2E1, as is discussed in the next article section

The second-line antituberculosis drug bedaquilineis mainly metabolized by N-demethylation via CYP3A4, and less so via CYP1A1, CYP2C8, CYP2C18, and CYP2C19 [151,152]. Bedaquiline is metabolized to N-desmethyl-bedaquiline, N-didesmethyl-bedaquiline, and two hydroxyl metabolites [153,154]. Another study showed N-dealkylation of bedaquiline to produce the aldehyde metabolite M5, primarily mediated by CYP3A4 [152,155]. Bedaquiline has a novel mechanism of action towards mycobacterial ATP synthase [155]; however, despite being highly effective it has been linked to adverse cardiac and hepatic drug reactions [155,156,157]. Recently, another second-line antituberculosis drug linezolid, has been shown to be metabolized via CYP2J2 and CYP1B1, by 2-hydroxylation and de-ethyleneation of the morpholine moiety of linezolid [158] It has been suggested that the less studied CYP2J2 accounts for approximately 50% of linezolid hepatic metabolism [158]. The second-line antituberculosis drug delamanid is mostly metabolized by albumin and to a lesser extent by CYP3A4. The entire metabolic profile of delamanid has not yet been uncovered [159].

## 5. Inhibition and/or Activation of CYP Enzymes by Drugs, and Drug–Drug Interactions

As seen earlier, CYP enzymes constitute the most important contributors to oxidative metabolism of drugs, and their inhibited and/or induced activity is an important determinant of several drug–drug interactions [160]. Some of the presently available antimalarial and antitubercular drugs have been observed to inhibit or induce human CYP enzymes which may cause drug–drug interactions that could either cause adverse drug reactions or decrease the efficacy of the drugs metabolized by these enzymes [2,3,141,161,162].

Recently, OZ439, a potent synthetic ozonide that is currently used for the treatment of uncomplicated malaria was found to inhibit CYP3A4 through both direct and mechanism-based inhibition [163]. Since CYP3A4 is involved in the metabolism of several antimalarial drugs such as artemisinins and their derivatives, chloroquine, dapsone, halofantrine, lumefantrine, mefloquine, primaquine, and quinine (Table 1), a combination therapy involving OZ439 and any of these drugs may not be advisable. Moreover, chloroquine has been implicated in decreased activity of CYP2D6, suggesting autoinhibition of its metabolism by this enzyme, while its less toxic derivative, hydroxychloroquine, also inhibited the enzyme significantly [113]. More evidence over the years have suggested that artemisinin and its relative drugs dihydroartemisinin, artesunate and artemether inhibit CYP1A2, 2B6, 2C19, and 3A4 [113,160,164,165,166,167]. CYP1A2 and CYP2C19 were noticeably inhibited by artemisinin and dihydroartemisinin, and in healthy individuals, CYP2D6 activity was 66% inhibited in vivo by artemisinin [160,167,168]. The activity of CYP2B6 was also reported to be inhibited by artemisinin derivatives in vitro [164,169]; however, inhibition by artemisinin itself proved to be weak [164]. Thus, while some artemisinins may be autoinhibitive to one of their metabolising enzymes CYP2B6, a combination of artemisinins with either mefloquin, primaquin, chloroquine, or proguanil may prove counterproductive.

Induction of human CYP enzymes has also been described, with evidence of artemisinin as a potent inducer of CYP1A2, CYP2A6, and CYP3A4 transcription and activity [164]. Artemisinin induces CYP enzyme expression, by activating two closely related nuclear hormone receptors responsible for the transcriptional regulation of CYP enzyme expression [170,171]. These are the pregnane X receptor (PXR) and the constitutive androstane receptor (CAR) which undergo either separate or cooperate induction by the artemisinin [170,171].

Like with antimalarial drugs, antituberculosis drugs such as isoniazid, rifampicin and ciprofloxacin have also been implicated in the inhibition or induction of CYP enzymes. Isoniazid is an inhibitor of CYP enzymes CYP1A2, CYP2A6, CYP2C9, CYP2C19, CYP2E1, and CYP3A4 [161], while ciprofloxacin inhibits CYP1A2, CYP2D6, and CYP3A4, and because of this inhibition, metabolism by these CYP enzymes is slowed down and a buildup of their substrates within the body occurs [2,3,161,172]. This is particularly unfavorable, as enzyme inhibition can cause potentially serious adverse events [3,161]. Administering isoniazid and ciprofloxacin alongside antimalarials such as mefloquin, primaquin, artemisinin, and all the CYP3A4 metabolized drugs highlighted above may result in adverse reactions.

In contrast to isoniazid and ciprofloxacin, rifampicin is a well-known inducer of several CYP enzymes in the CYP2A, CYP2B, CYP2C, and CYP3A family subgroups, but evidence has highlighted the high induction of CYP3A4 specifically [2,161]. The induction of these CYP enzymes results in an increased elimination of administered drugs, and this often results in reduced pharmacological effects [2,172]. For instance coadministration of amodiaquine and rifampicin in healthy volunteers resulted in significant decreases in the critical pharmacokinetic parameters of the drug, as opposed to increases in those of the main metabolite desethylamodiaquine, leading to a significant increase in the metabolic ratio from 1.55 to 2.68 [173]. No other TB drugs have been documented to inhibit or induce the CYP enzyme system; however, there were some investigations carried out on pyrazinamide and ethionamide to identify any inhibition of CYP enzymes by these drugs [174]. The results from that study revealed that although the three drugs (isoniazid, pyrazinamide and ethionamide) are closely related chemically, they do not all inhibit CYP enzymes to the same degree and thus pyrazinamide and ethionamide were disregarded as inhibitors of CYP enzymes [174].

**Figure 4:**
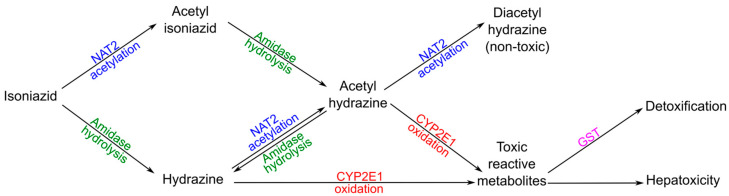
Human pathways of isoniazid metabolism involving NAT2, CYP2E1, and GST enzymes.

Isoniazid and its metabolic intermediates are regarded as the major source of hepatotoxicity in TB patients [175,176]. Isoniazid goes through hepatic metabolism by the N-acetyltransferase (NAT) enzyme system [177,178], being acetylated by NAT to acetyl-isoniazid and then biotransformed by hydrolysis via amidase to monoacetylhydrazine [2,179] (Figure 4). Monoacetylhydrazine is associated with hepatotoxicity and the most important detoxifying step to prevent this is a further acetylation step that produces the non-toxic product diacetylhydrazine [180,181]. However, in slow acetylators, extra monoacetylhydrazine is thought to be oxidized by CYP2E1 to toxic reactive metabolites instead of conversion to non-toxic diacetylhydrazine via NAT [182]. Moreover, when acetylation of isoniazid is slow it will be biotransformed to the toxic compound hydrazine and then most likely oxidized by CYP2E1 to produce reactive acetyl onium ions and acetyl radicals leading to hepatotoxicity [175,183,184]. Although probable, there is currently no explicit evidence of CYP2E1 oxidizing hydrazine and monoacetylhydrazine [185,186,187]. The enzyme glutathione s-transferase (GST) is an intracellular free radical scavenger that detoxifies the toxic reactive metabolites generated from antituberculosis drugs and other xenobiotics. Therefore, individuals with slower NAT2 activity, faster CYP2E1 activity, and slower GST activity are the most likely to experience hepatotoxicity from antituberculosis drugs [141,188,189,190,191]. Additionally, because rifampicin induces CYP2E1 [192] and isoniazid hydrolases [193], the incidence of hepatotoxicity has been found to be higher when isoniazid is combined with rifampicin.

The complexity increases when the HIV antiretroviral drug efavirenz is introduced. Efavirenz is transformed into inactive metabolites by CYP2B6, and less so by CYP2A6 [194]. Rifampicin induces CYP2B6 and should decrease efavirenz in the plasma [195]. Contrarily, a few patients prescribed with rifampicin and isoniazid have seen their efavirenz plasma levels increase (especially patients with slower activity CYP2B6 and NAT2 genotypes) [196,197,198]. This could be due to high isoniazid concentrations inhibiting CYP2A6 in NAT2 slow acetylators, as CYP2A6 activity is important for efavirenz clearance in CYP2B6 slow metabolizers [196,198,199].

## 6. Polymorphism in CYP Enzymes with Specific Focus on Antimalarial and Antituberculosis Drugs

The genes coding for CYP enzymes are highly polymorphic across populations, and this might lead to different enzyme activities impacting the efficacy of the drugs that they metabolize. The enzyme activity might increase, decrease or becomes nonexistent in some cases [7,11]. CYP gene variation engenders phenotypes classified as ultra-rapid, extensive, intermediate and poor metabolizers [35]. Extensive metabolizers have two “normal activity” alleles, generally called the *1 or consensus/reference allele. An ultra-rapid metabolizer usually has duplicated or multi-duplicated gene copies of the same allele (although this may not always be the case), intermediate and poor metabolizers have one and two defective alleles (e.g., gene inactivation or deletion), respectively [200,201,202].

CYP families 2 and 3 are implicated the most in terms of differences in metabolism due to variants. For example, a small study of adverse effects of CYP2A6 alleles and the antimalarial prodrug artesunate showed considerably higher adverse effects in patients with CYP2A6*1B variants [127]. CYP2A6*1B has a known ultra-rapid metabolism phenotype, which suggests that these patients experience adverse effects because of the accumulation of the active metabolite dihydroartemisinin. The polymorphism of CYP2C8 has resulted in missense mutations in alleles CYP2C8*2 and CYP2C8*3 that have been widely studied [133]. CYP2C8*2 occurs more frequently in African populations, while CYP2C8*3 is more frequent in Caucasians. Both of these alleles of CYP2C8 have been associated with slower metabolism, where there is a 50% reduction in enzyme activity in CYP2C8*2 and an 85% activity reduction in CYP2C8*3, as compared with the wild type/reference allele [203]. CYP2C8 along with CYP2B6, CYP3A4, and CYP3A5 metabolize drugs used in artemisinin-based combination therapy for malaria, and alleles such as CYP2C8*2, CYP2C8*3, CYP2B6*6, CYP3A4*1B, and CYP3A5*3 may affect patient response to this treatment [21]. The presence of alleles CYP2B6*6 and CYP3A4*1B vary significantly between African populations: 22% to 51% and 50% to 80%, respectively [204]. CYP2B6*6 is linked to a poor metabolizer phenotype for the antimalarial drug artemether [205]. Chloroquine is mostly metabolized by CYP2C8, but also by CYP2C9 and CYP3A5. The genetic variants of these enzymes may influence chloroquine pharmacokinetics [128]. For example, it has been seen that persons having “normal” CYP2C8 alleles (extensive metabolizers) have fewer gametocytes compared to persons with reduced-activity CYP2C8 alleles (*2, *3, and *4). CYP2C19 is known to metabolize proguanil and chlorproguanil, and its alleles CYP2C19*2 and CYP2C19*3 are largely associated with poor metabolism of these antimalarial drugs [111]. On the other hand, the CYP2C19*17 allele increases CYP2C19 expression and activity. Regarding treatment with the drug primaquine, there is evidence of patients with CYP2D6 poor-metabolizer alleles (*2, *4, *5, *10, *17, and *41) having therapeutic failure [206]. Lastly, CYP3A4 and CYP3A5 enzymes with poor-metabolizer phenotypes (CYP3A4*22, CYP3A5*3, CYP3A5*6, and CYP3A5*7) have been linked with quinine adverse reactions [207,208,209]. The impact of CYP genetic variants on antimalarial drugs and their clinical implications has also been reviewed in a recent article by Soyinka et al. 2022 [210].

With regards to TB, clearance of bedaquiline was 52% faster in Africans using a population pharmacokinetic model [211], which may be due to Africans expressing significantly more CYP3A5 compared to other populations [212]. Although bedaquiline is metabolized by CYP3A4, substrate specificity frequently overlaps between CYP3A4 and CYP3A5. A recent study found that in South Africans treated for MDR-TB, CYP3A5*3 was linked to slower bedaquiline clearance. The CYP3A5*3 allele results in nonfunctional CYP3A5 protein [213,214]. Several review articles mention that in East Asian populations, CYP2E1 RsaI/PstI polymorphisms are linked to an elevated risk of developing antituberculosis drug-induced liver injury [3,215,216,217,218]. In patients having the CYP2E1 RsaI/PstI c1/c1 genotype, isoniazid has a lower inhibitory effect on CYP2E1 activity compared to other genotypes, and therefore these patients have an increased chance of extra hepatotoxin production which then causes liver injury [216,218,219]. Moreover, the CYP2E1 rs2031920 variant genotype increases CYP2E1 activity, creating extra hepatotoxic metabolites of antituberculosis drugs (especially isoniazid) [220,221]. Conversely, some studies have found no significant connection between CYP2E1 genotype and antituberculosis drug-hepatoxicity [222,223]. In addition to CYP2E1, there may be a link between hepatotoxicity and polymorphisms in genes coding for CYP2C19: rs4244285 and rs4986893 polymorphisms may cause loss of gene functions and subsequently antituberculosis drug-induced liver injury [224]. Hypermethylation of CYP2E1 and CYP2D6 may increase the chance of liver injury from antituberculosis drugs as well [225,226].

### Afrocentric Missense Mutations

In line with high genetic diversity in the Continent compared to the rest of the world, CYP enzymes have also been shown to have greater diversity in Africa, possessing novel unique alleles [20,227,228]. Furthermore, comparative studies have identified population structuring at CYP genes, possibly associated with intra-African differences in response to drug therapies as well as the high rates of adverse drug reactions registered in the Continent [20,204,229]. This highlights the need for optimization of drug therapy and drug development in Africa.

**Figure 5:**
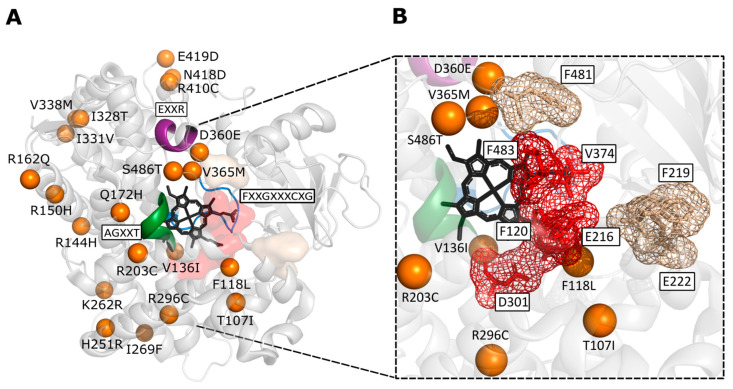
Afrocentric missense mutations and their location within the general CYP enzyme structure. (**A**) Mutations more frequent in African populations and highly restricted to African populations mapped to CYP2D6 (PDB ID 3TDA). Mutations in all Afrocentric alleles were mapped to their corresponding positions in CYP2D6 and colored orange. The (A/G)XXT region is shaded green, while the EXXR region is shown in the color purple, and the FXXGXXXCXG conserved domain is in blue. (**B**) Mutations in close proximity to known important residues in the active site and in the substrate channel. Active site residues of CYP2D6 shown in red, and residues in the substrate channel shown in cream. Mutation I19L could not be mapped to CYP2C19*15 as this region is not available on all crystalized CYP enzymes to date.

All CYP alleles related to the CYP enzymes in families 1, 2, and 3 that metabolize antimalarial and antituberculosis drugs were obtained from the PharmVar website (https://www.pharmvar.org/genes; accessed on 17 November 2022) in November 2022, and a full list of the alleles has been provided in Table S2. Of these, the alleles with the highest frequency in African populations and/or the alleles highly restricted to African populations, according to the recent comprehensive study by Zhou and Lauschke 2022 [230], were chosen from the list (Figure 2 and Table 2). The highest frequency being the allele in each sub-family that occurs most frequently in Africans (besides the reference allele), and highly restricted being the alleles that occur in Africans at least 4x the frequency of any other population type. These Afrocentric alleles were mapped to CYP enzyme structure (Figure 5), and a documentation of their known structural effects and functional consequences are provided (Table 2). A high proportion of Africans possess CYP2D6 alleles that are almost exclusive to the African population. A few notable alleles highly restricted to Africans are CYP2A6*17 (10.9%; second highest frequency of only 0.9% in Middle Eastern population), CYP2B6*18 (7%; second highest frequency of only 0.6% in Middle Eastern population), CYP2C9*9 (7.5%; second highest frequency of only 0.9% in Middle Eastern population), CYP2D6*17 (20.5%; second highest frequency of only 0.7% in admixed Americans), and CYP2D6*29 (8.9%; second highest frequency of only 0.4% in admixed Americans). The admixed American individuals with these CYP2D6 alleles are most likely African Americans. Moreover, worth mentioning, CYP2A6 alleles *23, *25, and *28 are almost exclusively found in Africans. All allele frequencies across all populations are available in the article by Zhou and Lauschke, 2022 [230].

A visual representation of where these 22 missense mutations are located on the general CYP structure is shown in Figure 5. Two of these Afrocentric missense mutations are situated near the catalytically important region that contains the conserved glutamic acid and arginine (EXXR) and decrease enzyme activity. As stated earlier, these two residues build salt bridge interactions which form the final tertiary structure of CYP enzymes [39]. The CYP2C9*5 missense mutation D360E is situated two residues away from the conserved EXXR region (Table 2) and was shown in molecular dynamics simulations to break the hydrogen bond between D360 and S478 (helix K and loop β4) leading to local structure destabilization [239]. Both aspartic acid and glutamic acid are negatively charged residues; however, glutamic acid is larger than aspartic acid. The other mutation, V365M from CYP2A6*17, forms part of the CYP2A6 active site region [231] and is four residues away from the conserved EXXR signature region (Figure 5A). Both valine and methionine are non-polar hydrophobic residues; however, methionine is larger and contains a sulfur atom [231]. The most frequent and restricted Afrocentric allele, CYP2D6*17 (20.5%; second highest frequency of only 0.7% in admixed Americans), contains mutation T107I that is located in substrate recognition site 1 (B′-helix) and causes changes in hydrogen bonds with surrounding residues within the active site cavity [247]. The hydrophilic threonine has an alcoholic side chain pointing down towards heme, whereas the hydrophobic isoleucine has a long aliphatic side chain points away from active site [246]. This CYP2D6*17 allele decreases enzyme activity, most likely due to the T107I mutation, as allele CYP2D6*2 contains the same mutations as CYP2D6*17 except for T107I but results in normal activity. Very concerning is allele CYP2B6*18, which is the only commonly found CYP2B6 missense mutation that causes enzyme inactivation [236,237], and it is highly restricted to Africans [230]. This allele contains mutation I328T (non-polar hydrophobic isoleucine to polar hydrophilic threonine) that is located in the substrate binding region. Another important Afrocentric allele, CYP2D6*29 (decreased enzyme function), contains substrate-binding region mutations V136I and V338M, with V136I also possibly contributing to cytochrome P450 reductase binding [245,248]. Expression studies reveal that both the V136I and the V338M mutations affect catalytic activity, and the effect is stronger when present together as seen in CYP2D6*29 [249].

## 7. Conclusions and Future Perspectives

Cytochrome P450 (CYP) enzyme families constitute the main pharmacogenes of humans, among which families 1, 2, and 3 are implicated in the phase I metabolism of most therapeutic or clinical drugs. An understanding of the mechanism of action of these enzymes is invaluable to the advancement of precision medicine. This review covers current knowledge on CYP enzymes in general, and more specifically on the first three CYP enzyme families with a focus on prevalent African alleles, particularly linking to two infectious diseases of poverty—malaria and tuberculosis—of which Africa has the highest burden.

Afrocentric alleles that may affect malaria treatment and the implicated antimalarials include: CYP2A6*17, CYP2A6*23, CYP2A6*25, CYP2A6*28 (artemisinin, artesunate), CYP2B6*6, CYP2B6*18 (artemisinin, artesunate, arteether), CYP2C8*2 (chloroquine), CYP2C9*5, CYP2C9*8, CYP2C9*9 (dapsone), CYP2C19*9, CYP2C19*13, CYP2C19*15 (dapsone, quinine, proguanil), CYP2D6*2, CYP2D6*17, CYP2D6*29 (chloroquine), and CYP3A4*15 (arteether, artemisinin, artemether, artelinic acid, chloroquine dapsone, halofantrine, lumefantrine, mefloquine, primaquine, quinine, quinidine).

Although there are reports on CYP enzymes that are involved in the metabolism of different antimalarial drugs, currently there is no literature regarding if first-line antituberculosis drugs are metabolized by any CYP enzymes. However, two second-line antituberculosis drugs bedaquiline and linezolid are known to be metabolized by CYP enzymes; the former mostly by CYP3A4 and the latter by CYP2J2 and CYP1B1. On another note, the first-line antituberculosis drug isoniazid is a known inhibitor while rifampicin is an inducer of several CYP enzymes, resulting in altered pharmacological effects of drugs that are metabolized by these CYPs. Further, variants of CYP2E1 and CYP2C19 have been implicated in altered isoniazid metabolism. Additionally, drug–drug interactions that influence the metabolism of antituberculosis, antimalarial, and other drugs, were explored.

As a first step to understand the mechanism by which these SNPs influence enzyme function, identified Afrocentric missense mutations were mapped to a CYP structure, and their known structural effects and functional consequences tabled. These afrocentric missence mutations indicate that they might play an important role in altering the metabolism of antimalarials amodiaquine, arteether, artelinic acid, artemether, artemisinin, artesunate, chloroquine, dapsone, halofantrine, lumefantrine, mefloquine, primaquine, proguanil, and quinine, as well as antituberculosis drugs bedaquiline and delamanid and thus need to be investigated further.

Several studies that have investigated the mechanism by which some CYP enzyme SNPs influence enzyme activity were covered in this review. These studies point to structural changes due to mutations as a major cause of the observed differences in the metabolism of these enzymes. Detailed structural studies will be required to fully elucidate the mechanisms by which these SNPs alter enzyme activity—with implications on precision medicine—especially as new mutants and phenotypic data are becoming available.

## Figures and Tables

**Table 1:** Some CYP enzyme metabolized antimalarial drugs and their oxidized products.

Antimalarial Drug	CYP Enzyme Metabolizer	Mechanism of Action	References
Amodiaquine	CYP2C8	Metabolised to desethylamodiaquine (DEAQ)—likely to proceed in two steps, a hydrogen abstraction and hydroxylation at the adjacent carbon, forming an unstable carbinolamide that rapidly hydrolyzes to DEAQ and acetaldehyde.	[116]
Arteether	CYP2B6, CYP3A4, CYP3A5	Arteether is deethylated to dihydroartemisinin (DHA), the main bioactive metabolite of atermisinin and its derivatives.	[120]
Artelinic acid	CYP3A4, CYP3A5	Artelinic acid is O-debenzylated to DHA	[120,121]
Artemether	CYP3A4, CYP1A2, CYP2B6	Artemether is demethylated to the bioactive metabolite DHA	[122,123,124]
Artemisinin	CYP2A6, CYP2B6, CYP3A4	Artemisinin is not itself metabolized to DHA but acts as the primary antimalarial. Upon reaction with Fe^2+^ it is converted first into oxygen centered free radicals derived by reductive cleavage of its peroxide bridge, which are then converted into carbon centered free radicals by intramolecular hydrogen abstraction from CH_2_ groups on the periphery of the artemisinin by the O centered radicals.	[116,125,126]
Artesunate	CYP2A6, CYP2B6	Rapidly hydrolyzed to the bioactive metabolite DHA	[116,125,126,127]
Chloroquine	CYP2C8, CYP2C19, CYP3A4, CYP2D6, CYP3A4, CYP3A5	Chloroquine is dealkylated into N-desethylchloroquine (DCQ) and N-bis-desethylchloroquine (BDCQ), with DCQ being the major metabolite.	[113,128,129]
Dapsone	CYP2C9, CYP3A4	Unlike other antimalarials, the first metabolizing enzyme of dapsone is N-acetyltransferase which hydrolyses the drug to the active form monoacetyl dapsone. CYP enzymes on the other hand hydrolyze the drug to its N-hydroxy metabolites dapsone hydroxylamine and monoacetyl hydroxylamine which are harmful hemolytic metabolites.	[116,117,119,130,131]
Halofantrine and Lumefantrine	CYP3A4, CYP3A5	Halofantrine undergoes desbutylation to N-desbutyl-halofantrine which possesses some antimalarial activity while lumefantrine is metabolized to desbutyl-lumefantrine and excreted via bile and faces.	[115,132]
Mefloquine	CYP1A2, CYP3A4	Metabolized into carboxymefloquine metabolite which has little or no antimalarial activity.	[121,133,134,135]
Primaquine	CYP1A2, CYP3A4, CYP2D6	Three possible pathways exist for primaquine metabolism and that involving CYP enzymes is hydroxylation at different positions on the quinoline ring, with mono-, di-, or even tri-hydroxylations possible, and subsequent glucuronide conjugation of the hydroxylated metabolites. The main metabolite carboxyprimaquine comes about through a monoamine oxidase catalyzed oxidative deamination.	[116,136]
Proguanil	CYP2C19	Oxidative metabolized to cycloguanil, which is the active form of the drug	[116,121,137]
Quinine	CYP3A4	Undergoes hydroxylation to the main metabolite 3-hydroxiquinine	[28,121,138,139]

**Table 2:** CYP alleles with missense mutations that have the highest frequency in African populations and/or are highly restricted to African populations [18,230]. Known structural effects and functional consequences of the mutations are included.

CYP Enzyme	Antimalarial/Antituberculosis Drug Metabolized	Allele	Frequency in African Populations (%)	Amino acid Mutation Position	Functional Consequence	Residue/Mutation Notes
CYP2A6	Artemisinin, artesunate	CYP2A6*17	10.9	V365M	-	Located in CYP2A6 substrate recognition site SRS-5 forming part of the active site region and some side chains that point into the heme pocket, suggesting this residue may be important for substrate specificity. In vitro enzyme assays and metabolism studies showed no effect of the mutation on the stability of the enzyme. Both valine and methionine are non-polar hydrophobic residues; however, methionine is larger and contains a sulfur atom [231].
		CYP2A6*23	1.4	R203C	Decreased	Residue located on α-helix F in substrate recognition site 2/3. Change from large basic residue to medium sized polar residue. Molecular modeling suggests Arg203 could orient important binding residue Phe209 [232,233].
		CYP2A6*25	1.4	F118L	-	During molecular dynamics simulation, the F118L mutant side chain moves away from the heme and affects secondary structure formation and interaction with heme and substrates [234].
		CYP2A6*28	1.5	N418D E419D	-	N418D and E419D cause a structural change in the substrate access channel and the substrate binding site [234].
CYP2B6	Artemisinin, artesunate, arteether	CYP2B6*6	32	Q172H; K262R	Decreased	Q172H and K262R are not located at the active site and have not been identified in substrate recognition sites [235].
		CYP2B6*18	7	I328T	Inactive	Results in no detectable protein or activity in vitro [236]. Designated as a null allele [237]. The only commonly found inactive CYP2B6 missense mutation, and highly restricted to Africa [230].
CYP2C8	Chloroquine, amodiaquine, bedaquiline	CYP2C8*2	15.2	I269F	Decreased	Residue located on enzyme surface. Larger residue change. Possible effect on enzyme folding and interaction with cytochrome P450 reductase [238].
CYP 2C9	Dapsone	CYP 2C9*5	1.1	D360E	Decreased	In molecular dynamics simulations, D360E broke the hydrogen bond between D360 and S478 (helix K and loop β4) leading to local structure destabilization. Glutamic acid is larger than aspartic acid [239].
		CYP 2C9*8	6	R150H	Decreased	R150 is highly conserved. Located on protein surface at D-helix region, away from the active site. The structure of the CYP2C9*8-losartan complex has ~60° rotation of the H150 sidechain in an alternate conformation compared to the sidechain of R150 [240]. Possible involvement of R150H in the salt bridge network with the neighboring residues, suggesting the change to histidine at this solvent-exposed region may no longer coordinate similar ionic and electrostatic interactions and may result in destabilization of the structure [241]. Possible that this change could influence reductase binding [242].
		CYP 2C9*9	7.5	H251R	Normal	H251R is located near the C-terminal end of helix G and hydrogen bonds to D262 on helix H. Arginine has a longer side chain than histidine [242].
CYP 2C19	Proguanil, quinine, dapsone, bedaquiline	CYP 2C19*9	1.3	R144H I331V	Decreased	The R144H mutation could affect enzyme structure and function. The conserved arginine is located in the D helix and is part of a complex salt bridge with conserved Ser180 in the E helix and the backbone of the turn before helix H. The arginine could help stabilize the structure of the enzyme and may also be a part of the hinge for the F–G loop [243]. Docking experiments suggest that I331 is involved in ligand binding. The I331V mutant has differing lipophilicity in the binding pocket or active cavity [239,244].
		CYP 2C19*13	1.8	I331V R410C	Normal	Residue 410 located on enzyme surface. Change from large basic residue to medium sized polar residue. Mutation has neutral effect on enzyme activity.
		CYP 2C19*15	1.9	I19L I331V	Normal	The I19L mutation with residue 19 is part of signal-anchor sequence, located in truncated N-terminal region. Mutation has neutral effect [245].
CYP 2D6	Chloroquine	CYP 2D6*2	22.5	R296C S486T	Normal	R296C is located in substrate recognition site 4 (I-helix) important for catalytic proton delivery. Kinetics data indicate that R296C in CYP2D6*2 causes enhanced ligand binding possibly due to morphological changes Change from medium-sized basic residue to small polar residue. [246]. S486T mutation is located in substrate recognition site (β4-2 sheet) and is very close to active site and heme [239]. Kinetics data indicate that S486T in CYP2D6*2 causes enhanced ligand binding possibly due to morphological changes [246].
		CYP 2D6*17	20.5	T107I R296C S486T	Decreased	T107I is located in substrate recognition site 1 (B′-helix). Substitution of hydrophilic Threonine to hydrophobic Isoleucine. Threonine has alcoholic side chain pointing down towards heme, whereas Isoleucine long aliphatic side chain points away from active site [246]. Causes changes in hydrogen bonds with surrounding residues within the active site cavity [247].
		CYP 2D6*29	8.9	V136I R296C V338M S486T	Decreased	V136 is located between substrate recognition sites 1 and 2 in helix C [248]. In contact with cytochrome P450 reductase. Neutral effect on protein stability [245]. V338M is located between substrate recognition sites 4 and 5 at the end of helix J [248]. Neutral effect on protein stability [245]. Expression studies reveal that both the V136I and the V338M mutations affect catalytic activity, and the effect is stronger when present together as seen in CYP2D6*29. Both valine and methionine are non-polar hydrophobic residues; however, methionine is larger and contains a sulfur atom [249].
CYP3A4	Quinine, quinidine, chloroquine, mefloquine, primaquine, halofantrine, lumefantrine, dapsone, artemisinin, artemether, arteether, artelinic, acid, delaminid, bedaquiline	CYP3A4*15	2.6	R162Q	-	Mutation related to rapid metabolism of quinine in vitro. Change from large basic residue to medium-sized polar hydrophilic residue [250].

## Data Availability

No new data were created or analyzed in this study. Data sharing is not applicable to this article.

## Supplementary Materials

The following supporting information can be downloaded at: https://www.mdpi.com/article/10.3390/ijms24043383/s1.
