# Supplementary material for: Human Cytochrome P450 1, 2, 3 Families as Pharmacogenes with Emphases on Their Antimalarial and Antituberculosis Drugs and Prevalent African Alleles
Source: Int J Mol Sci. 2023 Feb 8;24(4):3383. doi: 10.3390/ijms24043383 (PMC9961538; doi:10.3390/ijms24043383)
Supplement: Supplementary file 1 [file ijms-24-03383-s001.zip › ijms-2130595-supplementary.pdf]

Supplementary Material

Human cytochrome P450 1, 2, 3 families as pharmacogenes with emphases on their antimalarial and antituberculosis drugs and prevalent African alleles

Chiratidzo R. Chamboko[1,†], Wayde Veldman[1,†], Rolland Bantar Tata[1], Birgit Schoeberl[2] and Özlem Tastan Bishop[1,*]

[1]Research Unit in Bioinformatics (RUBi), Department of Biochemistry and Microbiology, Rhodes University, Makhanda/Grahamstown 6139, South Africa

[2]Novartis Institutes for BioMedical Research PK Sciences / Translational Medicine 220 Massachusetts Ave, Cambridge, MA 02139, USA

[†] Equally contributed first authorship

*Corresponding author: Özlem Tastan Bishop (o.tastanbishop@ru.ac.za)

KEYWORDS: sequence and structure analysis; CYP metabolism; polymorphisms; SNPs; missense mutations; personalized medicine

1. Supplementary Figures

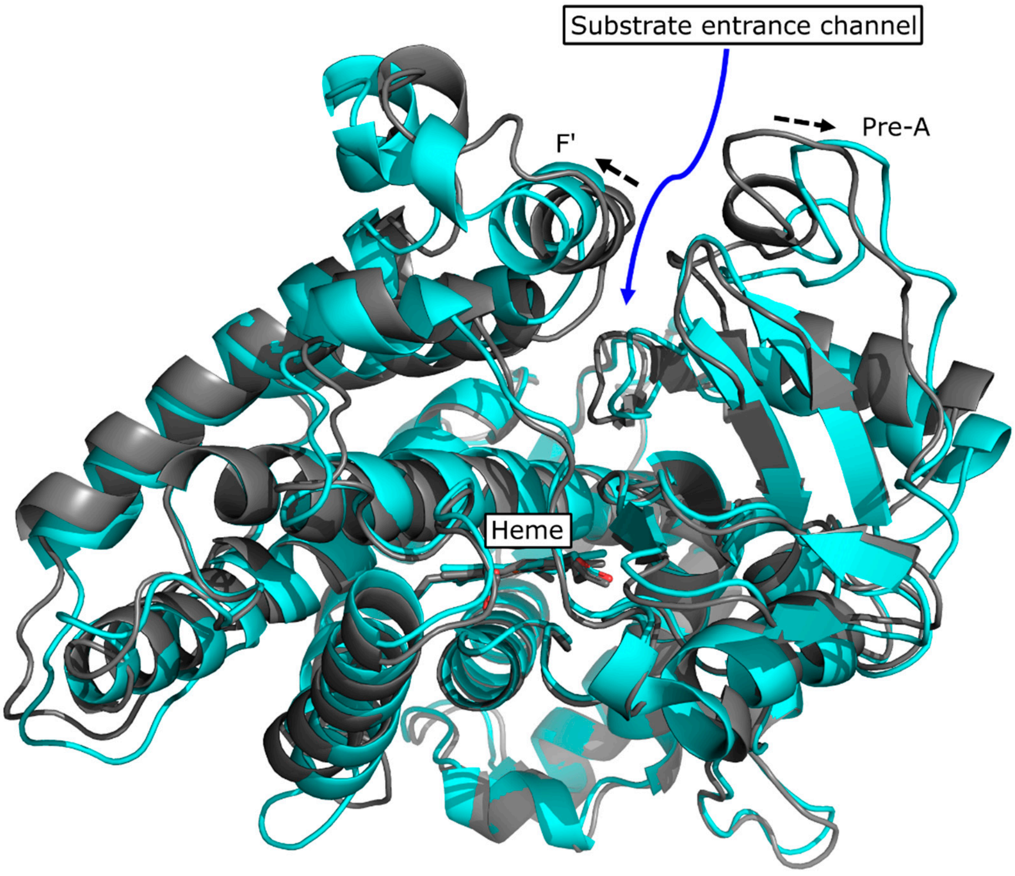

Figure S1: Open and closed conformations of CYP2D6. Open conformation of CYP2D6 structure in cyan from PDB (3TBG) superimposed onto closed conformation structure in grey (3QM4). Direction of displacement of F' and Pre-A regions to form the open structure is shown in dotted arrows.

2. Supplementary Tables

Table S1: 3D structures of enzymes from CYP 1, 2 and 3 enzyme families that are available on PDB with their respective resolutions and co-crystalized ligands as of November 2022.

Family	Enzyme name	PDB IDs	Resolution (Å)	Co-crystalised ligand
**CYP 1**	CYP 1A1	4I8V	2.60	Alpha-naphthylflavone
		6DWM	2.85	Bergamottin
		6UDL	2.85	Duocarmycin Prodrug (S) ICT-2700
		6DWN	3	Erlotinib
		6UDM	3.07	Duocarmycin Prodrug (S) ICT-2700
		6O5Y	3.17	5-amino-N-{5-[(4R,5R)-4-amino-5-fluoroazepan-1-yl]-1-methyl-1H-pyrazol-4-yl}-2-(2,6-difluorophenyl)-1,3-thiazole-4-carboxamide
	CYP 1A2	2HI4	1.95	Alpha-naphthylflavone
	CYP 1B1	3PM0	2.70	Alpha-naphthylflavone
		6OYU	2.95	Alpha-naphthylflavone
		6OYV	3.10	Estradiol
		6IQ5	3.70	2-(cis-4-azidocyclohexyl)-4H-naphtho[1,2-b]pyran-4-one
**CYP 2**	CYP 2A6#	2FDV	1.65	N-Methyl(5-(pyridin-3-yl)furan-2-yl)methanamine
		2FDU	1.85	N,N-Dimethyl(5-(pyridin-3-yl)furan-2-yl)methanamine
		1Z10	1.90	Coumarin
		2FDY	1.95	Adrithiol
		2PG5	1.95	-
		2FDW	2.05	5-(Pyridin-3-yl)furan-2-yl)methanamine
		1Z11	2.05	Methoxsalen
		3T3Q	2.10	Pilocarpine
		3EBS	2.15	Phenacetin
		4EJJ	2.30	Nicotine
	CYP 2A7	-	-	-
	CYP 2A13	4EJI	2.10	4-(methylnitrosamino)-1-(3-puridyl)-1-butanone
		2P85	2.35	Indole
		4EJH	2.35	4-(methylnitrosamino)-1-(3-pyridyl)-1-butanone
		4EJG	2.50	Nicotine
		3T3S	3.00	Pilocarpine
	CYP 2B6#	5UFG	1.76	Myrtenyl bromide
		5UDA	1.93	Camphane
		4I91	2.00	Alpha-pinene
		3IBD	2.00	4-(4-chlorophenyl)imidazole
		5UAP	2.03	Bornyl bromide
		4RQL	2.10	Sabinene

		3QOA<br>4RRT<br>4ZV8<br>5UEC	2.10<br>2.20<br>2.24<br>2.27	4-Benzylpyridine<br>(+)-3-carene<br>Alpha-pinene<br>Myrtenyl bromide
	CYP 2C8	2NNJ<br>2NNH<br>1PQ2<br>2VN0<br>2NNI	2.28<br>2.60<br>2.70<br>2.70<br>2.80	Felodipine<br>9-cis-retinoic acid<br>-<br>Troglitazone<br>Montelukast
	CYP 2C9[#]	5X23<br>5W0C<br><br><br>1R9O<br>5A5I<br><br><br>7RL2<br>5XXI<br>5K7K<br><br><br>4NZ2<br><br><br>5X24<br>1OG5	2.00<br>2.00<br><br><br>2.00<br>2.00<br><br><br>2.23<br>2.30<br>2.30<br><br><br>2.45<br><br><br>2.48<br>2.55	Losartan<br>Ethyl {2-[([1,3]thiazolo[4,5-c]pyridine-2-carbonyl)amino]thiophene-3-carbonyl}carbamate<br>Flurbiprofen<br>N-[4-(3-chloranyl-4-cyano-phenoxy)cyclohexyl]-1,1,1-tris(fluoranyl)methanesulfonamide<br>Losartan<br>Losartan<br>4-[4-chloranyl-2-(1~{H}-pyrazol-4-yl)phenoxy]-3-cyano-~{N}-(1,3-thiazol-2-yl)benzenesulfonamide (2R)-N-{4-[(3-bromophenyl)sulfonyl]-2-chlorophenyl}-3,3,3-trifluoro-2-hydroxy-2-methylpropanamide<br>Losartan<br>S-warfarin
	CYP 2C18	-	-	-
	CYP 2C19	4GQS	2.87	(4-hydroxy-3,5-dimethylphenyl)(2-methyl-1-benzofuran-3-yl)methanone
	CYP 2D6[#]	3TBG<br>4WNU<br>4WNV<br>6CSD<br>6CSB<br>4XRZ<br><br><br>4XRY<br><br><br>4WNT<br>3TDA<br>5TFT	2.10<br>2.26<br>2.35<br>2.39<br>2.39<br>2.40<br><br><br>2.50<br><br><br>2.60<br>2.67<br>2.71	Thioridazine<br>Quinidine<br>Quinine<br>Prinomastat<br>Thioridazine<br>(4aR,6R,8aS)-8a-(2,4-difluorophenyl)-6-(1H-pyrazol-4-yl)-4,4a,5,6,8,8a-hexahydropyrano[3,4-d][1,3]thiazin-2-amine<br>(4aR,6R,8aS)-8a-(2,4-difluorophenyl)-6-(1-methyl-1H-pyrazol-4-yl)-4,4a,5,6,8,8a-hexahydropyrano[3,4-d][1,3]thiazin-2-amine<br>Ajmalicine<br>Prinomastat<br>(4S)-4-[2,4-difluoro-5-({[1-(trifluoromethyl) cyclopropyl]amino}methyl)phenyl]-4-methyl-5,6-dihydro-4H-1,3-thiazin-2-amine
	CYP 2E1	3E6I<br>3T3Z<br>3E4E<br>3GPH<br>3KOH	2.20<br>2.35<br>2.60<br>2.70<br>2.90	1H-indazole<br>Pilocarpine<br>4-methylpyrazole<br>Omega-imidazolyl-decanoic acid Omega-imidazolyl octanoic acid

		3LC4	3.10	Omega-imidazolyl-dodecanoic Acid
	CYP 2F1	-	-	-
	CYP 2J2	-	-	-
	CYP 2R1	3CZH<br>3DL9<br>3C6G	2.30<br>2.72<br>2.80	Vitamin D2<br>1-alpha-hydroxy-vitamin D2<br>Vitamin D3
	CYP 2S1	-	-	-
	CYP 2U1	-	-	-
	CYP2W1	-	-	-
**CYP 3**	CYP 3A4[#]	5VCC<br>6MA8<br>4D6Z<br><br><br>5VCD<br>3NXU<br>1TQN<br>6MA7<br>3UA1<br>6DAA<br><br><br>6MA6	1.70<br>1.83<br>1.93<br><br><br>1.95<br>2.00<br>2.05<br>2.09<br>2.15<br>2.15<br><br><br>2.18	-<br>Phenylmethylsulfonyl fluoride<br>Tert-butyl {6-oxo-6-[(pyridin-3-ylmethyl)amino]hexyl}carbamate and Imidazole<br>-<br>Ritonavir<br>-<br>Fluconazole<br>Bromoergocryptine<br>Tert-butyl [(2S)-1-{[(2R)-1-oxo-3-phenyl-1-{[(pyridin-3-yl)methyl]amino}propan-2-yl]sulfanyl}-3-phenylpropan-2-yl]carbamate<br>Metyrapone
	CYP 3A5	6MJM<br>7SV2<br>7LAD<br>5VEU	2.20<br>2.46<br>2.65<br>2.91	-<br>Azamulin<br>Clobetasol propionate<br>Ritonavir
	CYP 3A7	7MK8	2.15	Dithiothreitol
	CYP 3A43	-	-	-

[#] -CYP enzymes with more than 10 structures on PDB (for the table a cut-off of 10 structures with the best resolution was used).

Table S2: Cytochrome P450 enzymes from families 1, 2 and 3 associated with metabolism of antimalarial and antituberculosis drugs with their alleles and amino acid mutation positions.

CYP family	CYP enzyme	Antimalarial/antituberculosis drug affected	Allele name	Amino acid mutation position
**CYP 1**	CYP1A1	Bedaquiline	CYP1A1*4	T461N
			CYP1A1*5	R464S
			CYP1A1*6	M331I
			CYP1A1*8	I448N
			CYP1A1*9	R464C
			CYP1A1*10	R477W
			CYP1A1*11	P492R
			CYP1A1*12	A62P
			CYP1A1*13	G45D
	CYP1A2	Mefloquine, primaquine	CYP1A2*2	F21L
			CYP1A2*3	D348N
			CYP1A2*4	I386F
			CYP1A2*5	C406Y
			CYP1A2*6	R431W
			CYP1A2*8	R456H
			CYP1A2*9	T83M
			CYP1A2*10	E168Q
			CYP1A2*11	F186L
			CYP1A2*12	S212C
			CYP1A2*13	G299S
			CYP1A2*14	T438I
			CYP1A2*15	P42R
			CYP1A2*16	R377Q
			CYP1A2*17	T395M
			CYP1A2*18	N397H
			CYP1A2*19	R510Q
			CYP1A2*20	D436N
	CYP1B1	Linezolid	CYP1B1*2	R48G; A119S
			CYP1B1*3	L432V
			CYP1B1*4	N453S
			CYP1B1*5	R48G; L432V
			CYP1B1*6	R48G; A119S; L432V
			CYP1B1*7	R48G; A119S; L432V; A443G
			CYP1B1*8	L432V; D441H
			CYP1B1*11	W57C
			CYP1B1*12	G61E
			CYP1B1*18	G365W
			CYP1B1*19	P379L
			CYP1B1*20	E387K

			CYP1B1*21	R390H
			CYP1B1*23	P437L
			CYP1B1*25	R469W
**CYP 2**	CYP2A6	Artemisinin, artesunate	CYP2A6*2	L160H
			CYP2A6*5	G479V
			CYP2A6*6	R128Q
			CYP2A6*7	I471T
			CYP2A6*8	R485L
			CYP2A6*10	I471T; R485L
			CYP2A6*11	S224P
			CYP2A6*13	G5R
			CYP2A6*14	S29N
			CYP2A6*15	K194E
			CYP2A6*16	R203S
			CYP2A6*17	V365M
			CYP2A6*18	Y392F
			CYP2A6*19	Y392F; I471T
			CYP2A6*23	R203C
			CYP2A6*24	V110L; N438Y
			CYP2A6*25	F118L
			CYP2A6*26	F118L; R128L; S131A
			CYP2A6*28	N418D; E419D
			CYP2A6*31	M6L
			CYP2A6*34	M6L; V13A; K30R; Q53H; M54I; Y55C; N56D; L58I; I61F; R64C; V117A; R128L; S131A; A153S; D158E; L160I; G162S; G164H; K194E; R203S; M204V
			CYP2A6*35	N438Y
			CYP2A6*36	N438Y; I471T
			CYP2A6*37	N438Y; I471T; R485L
			CYP2A6*38	Y351H
			CYP2A6*39	V68M
			CYP2A6*40	I149M
			CYP2A6*41	R265Q
			CYP2A6*42	I268T
			CYP2A6*43	T303I
			CYP2A6*44	E390K; N418D; E419D
			CYP2A6*45	L462P
	CYP2B6	Artemisinin, artesunate, arteether	CYP2B6*2	R22C
			CYP2B6*3	S259R
			CYP2B6*4	K262R
			CYP2B6*5	R487C
			CYP2B6*6	Q172H; K262R
			CYP2B6*7	Q172H; K262R; R487C
			CYP2B6*8	K139E

			CYP2B6*9	Q172H
			CYP2B6*10	Q21L; R22C
			CYP2B6*11	M46V
			CYP2B6*12	G99E
			CYP2B6*13	K139E; Q172H; K262R
			CYP2B6*14	R140Q
			CYP2B6*15	I391N
			CYP2B6*16	K262R; I328T
			CYP2B6*17	T26S; D28G; R29T
			CYP2B6*18	I328T
			CYP2B6*19	Q172H; K262R; R336C
			CYP2B6*20	T168I; Q172H; K262R
			CYP2B6*21	P428T
			CYP2B6*23	M459V
			CYP2B6*24	G476D
			CYP2B6*25	Q485L
			CYP2B6*26	P167A; Q172H; K262R
			CYP2B6*27	M198T
			CYP2B6*31	L313I
			CYP2B6*32	A407T
			CYP2B6*33	R487S
			CYP2B6*34	Q172H; K262R; R487S
			CYP2B6*35	G110V; I114T; E148D; M198T; A279P
			CYP2B6*36	Q172H; K262R
			CYP2B6*37	Q172H; V183G; K262R
	CYP2C8	Chloroquine, amodiaquine, bedaquiline	CYP2C8*2	I269F
			CYP2C8*3	R139K; K399R
			CYP2C8*4	I264M
			CYP2C8*6	G171S
			CYP2C8*8	R186G
			CYP2C8*9	K247R
			CYP2C8*10	K383N
			CYP2C8*13	I223M
			CYP2C8*14	A238P
			CYP2C8*15	V181I
			CYP2C8*16	I331T
			CYP2C8*17	I244V
			CYP2C8*18	L361F
	CYP2C9	Dapsone	CYP2C9*2	R144C
			CYP2C9*3	I359L
			CYP2C9*4	I359L
			CYP2C9*5	D360E
			CYP2C9*7	L19I
			CYP2C9*8	R150H
			CYP2C9*9	H251R
			CYP2C9*10	E272G
			CYP2C9*11	R335W

			CYP2C9*12	P489S
			CYP2C9*13	L90P
			CYP2C9*14	R125H
			CYP2C9*16	T299A
			CYP2C9*17	P382S
			CYP2C9*18	I359L; D397A
			CYP2C9*19	Q454H
			CYP2C9*20	G70R
			CYP2C9*21	P30L
			CYP2C9*22	N41D
			CYP2C9*23	V76M
			CYP2C9*24	E354K
			CYP2C9*26	T130R
			CYP2C9*27	R150L
			CYP2C9*28	Q214L
			CYP2C9*29	P279T
			CYP2C9*30	A477T
			CYP2C9*31	I327T
			CYP2C9*32	V490F
			CYP2C9*33	R132Q
			CYP2C9*34	R335Q
			CYP2C9*35	R125L; R144C
			CYP2C9*36	M1V
			CYP2C9*37	D49G
			CYP2C9*38	G96A
			CYP2C9*39	G98V
			CYP2C9*40	F110S
			CYP2C9*41	K119R
			CYP2C9*42	R124Q
			CYP2C9*43	R124W
			CYP2C9*44	T130M
			CYP2C9*45	R132W
			CYP2C9*46	A149T
			CYP2C9*47	P163L
			CYP2C9*48	I207T
			CYP2C9*49	I222V
			CYP2C9*50	P227S
			CYP2C9*51	I284V
			CYP2C9*52	T299R
			CYP2C9*53	P317S
			CYP2C9*54	S343R
			CYP2C9*55	L361I
			CYP2C9*56	I387V
			CYP2C9*57	N204H
			CYP2C9*58	P337T
			CYP2C9*59	I434F
			CYP2C9*60	L467P
			CYP2C9*61	R144C; N457S

			CYP2C9*62	R125C
			CYP2C9*63	R144H
			CYP2C9*64	S280C
			CYP2C9*65	E326D
			CYP2C9*66	L362V
			CYP2C9*67	R433W
			CYP2C9*69	D191G
			CYP2C9*70	G442V
			CYP2C9*71	E272G; P489S
			CYP2C9*72	A149V
			CYP2C9*73	R150C
			CYP2C9*74	Q214H
			CYP2C9*75	N418T
			CYP2C9*76	G40R
			CYP2C9*77	G96R
			CYP2C9*78	L128R
			CYP2C9*79	P346L
			CYP2C9*80	C13G
			CYP2C9*81	E300V
			CYP2C9*82	P227L
			CYP2C9*83	L36F
			CYP2C9*84	G431R
	CYP2C18	Bedaquiline	-	-
	CYP2C19	Proguanil, quinine, dapsone	CYP2C19*1	I331V
			CYP2C19*4	M1V; I331V
			CYP2C19*5	R433W
			CYP2C19*6	R132Q; I331V
			CYP2C19*8	W120R
			CYP2C19*9	R144H; I331V
			CYP2C19*10	P227L; I331V
			CYP2C19*11	R150H; I331V
			CYP2C19*13	I331V; R410C
			CYP2C19*14	L17P; I331V
			CYP2C19*15	I19L; I331V
			CYP2C19*16	R442C
			CYP2C19*17	I331V
			CYP2C19*18	R329H; I331V
			CYP2C19*19	S51G; I331V
			CYP2C19*22	R186P; I331V
			CYP2C19*23	G91R; I331V
			CYP2C19*24	I331V; R335Q
			CYP2C19*25	I331V; F448L
			CYP2C19*26	D256N; I331V
			CYP2C19*28	I19L; I331V; V374I
			CYP2C19*29	K28I; I331V
			CYP2C19*30	R73C
			CYP2C19*31	H78Y; I331V
			CYP2C19*32	H99R; I331V

			CYP2C19*33	D188N; I331V
			CYP2C19*34	P3S; F4L
			CYP2C19*39	I19L; E122A; I331V
	CYP2D6	Chloroquine	CYP2D6*2	R296C; S486T
			CYP2D6*7	H324P
			CYP2D6*10	P34S; S486T
			CYP2D6*12	G42R; R296C; S486T
			CYP2D6*14	G169R; R296C; S486T
			CYP2D6*17	T107I; R296C; S486T
			CYP2D6*22	R28C
			CYP2D6*23	A85V
			CYP2D6*24	I297L
			CYP2D6*25	R343G
			CYP2D6*26	I369T
			CYP2D6*27	E410K
			CYP2D6*28	V7M; Q151E; R296C; S486T
			CYP2D6*29	V136I; R296C; V338M; S486T
			CYP2D6*31	R296C; R440H; S486T
			CYP2D6*33	A237S
			CYP2D6*34	R296C
			CYP2D6*35	V11M; R296C; S486T
			CYP2D6*36	P34S; P469A; T470A; H478S; G479R; F481V; A482S; S486T
			CYP2D6*37	P34S; R201H; S486T
			CYP2D6*39	S486T
			CYP2D6*43	R26H
			CYP2D6*45	E155K; R296C; S486T
			CYP2D6*46	R26H; E155K; R296C; S486T
			CYP2D6*47	R25W; P34S; S486T
			CYP2D6*48	A90V
			CYP2D6*49	P34S; F120I; S486T
			CYP2D6*50	E156A
			CYP2D6*51	R296C; E334A; S486T
			CYP2D6*52	P34S; E418K; S486T
			CYP2D6*53	F120I; A122S
			CYP2D6*54	P34S; T261I; S486T
			CYP2D6*55	R296C; K404Q; S486T
			CYP2D6*57	P34S; R62W; P469A; T470A; H478S; G479R; F481V; A482S; S486T
			CYP2D6*62	R441C
			CYP2D6*64	P34S; T107I; S486T
			CYP2D6*65	P34S; R296C; S486T
			CYP2D6*70	V119M; V136I; V338M; S486T

			CYP2D6*71	G42E
			CYP2D6*72	P34S; E383K; S486T
			CYP2D6*73	V104M; R296C; S486T
			CYP2D6*74	L91M
			CYP2D6*75	R441H
			CYP2D6*82	L91M; H94R; V104A; T107Y; I109V
			CYP2D6*83	P469A; T470A; H478S; G479R; F481V; A482S
			CYP2D6*84	P267H; R296C; S486T
			CYP2D6*85	R296C; H478Q; S486T
			CYP2D6*86	E278K; M279K
			CYP2D6*87	A5V; P34S; S486T
			CYP2D6*88	V104A; S486T
			CYP2D6*89	L142S
			CYP2D6*90	K147R
			CYP2D6*93	T249P
			CYP2D6*94	P34S; D337G; S486T
			CYP2D6*95	P34S; R388H; S486T
			CYP2D6*97	F457L
			CYP2D6*98	R296C; H463D; S486T
			CYP2D6*99	P34S; R88P; S486T
			CYP2D6*102	A90V; R296C; S486T
			CYP2D6*103	A90V; N166D; R296C; S486T
			CYP2D6*104	E156V; R296C; S486T
			CYP2D6*105	R296C; F366S; S486T
			CYP2D6*106	E418K
			CYP2D6*107	V136M
			CYP2D6*108	H352R; Y355C
			CYP2D6*110	G445R
			CYP2D6*111	G111S; R296C; S486T
			CYP2D6*112	S488F
			CYP2D6*113	G439D
			CYP2D6*114	P34S; G169R; R296C; S486T
			CYP2D6*116	R330P
			CYP2D6*117	R296C; D337N; S486T
			CYP2D6*118	T310A
			CYP2D6*121	N166D; H167Q; S168A; R296C; S486T
			CYP2D6*122	V370I
			CYP2D6*125	R296C; R450H; S486T
			CYP2D6*126	S135F; R296C; S486T
			CYP2D6*127	Y355C; R365H
			CYP2D6*128	V68G; R296C; S486T
			CYP2D6*130	C191L
			CYP2D6*131	L231P

			CYP2D6*132	P34S; N285S; S486T
			CYP2D6*133	R296C; G340R; S486T
			CYP2D6*134	R344Q
			CYP2D6*135	R296C; R414C; S486T
			CYP2D6*136	R296C; P430L; S486T
			CYP2D6*137	R497H
			CYP2D6*139	R365H
			CYP2D6*140	L230F
			CYP2D6*141	T107I; R296C; E410K; P469A; T470A; H478S; G479R; F481V; A482S; S486T
			CYP2D6*142	R28C; P34S; S486T
			CYP2D6*146	R26H; R296C; S486T
			CYP2D6*148	R26H; R296C; E410K; S486T
			CYP2D6*149	D97E; V136I; R296C; V338M; S486T
			CYP2D6*150	L213P; R296C; S486T
			CYP2D6*151	N175S
			CYP2D6*152	A226V
			CYP2D6*153	E215K
			CYP2D6*154	T107I; R296C; R441H; S486T
			CYP2D6*155	V136I; R296C; V338M; M451I; S486T
			CYP2D6*157	V136I; A165V; R296C; V338M; S486T
			CYP2D6*159	P41L; R296C; S486T
			CYP2D6*160	L91M; H94R; R296C; S486T
			CYP2D6*162	R296C; 410K; S486T
			CYP2D6*163	W128R; R296C; S486T
			CYP2D6*164	V136I; S486T
			CYP2D6*165	V136I; R173H; R296C; V338M; S486T
			CYP2D6*167	Q117R
			CYP2D6*168	G42E; E196K
			CYP2D6*169	E402L
			CYP2D6*170	P430L
			CYP2D6*171	V119M; V136I; R296C; V338M; S486T
	CYP2J2	Linezolid	CYP2J2*2	T143A
			CYP2J2*3	R158C
			CYP2J2*4	I192N
			CYP2J2*5	D342N
			CYP2J2*6	N404Y
			CYP2J2*8	G312R
			CYP2J2*9	P351L

			CYP2J2*10	P115L
**CYP 3**	CYP3A4	Quinine, quinidine, chloroquine, mefloquine, primaquine, halofantrine, lumefantrine, dapsone, artemisinin, artemether, arteether, artelinic acid, bedaquiline, delaminid	CYP3A4*2	S222P
			CYP3A4*3	M445T
			CYP3A4*4	I118V
			CYP3A4*5	P218R
			CYP3A4*7	G56D
			CYP3A4*8	R130Q
			CYP3A4*9	V170I
			CYP3A4*10	D174H
			CYP3A4*11	T363M
			CYP3A4*12	L373F
			CYP3A4*13	P416L
			CYP3A4*14	L15P
			CYP3A4*15	R162Q
			CYP3A4*16	T185S
			CYP3A4*17	F189S
			CYP3A4*18	L293P
			CYP3A4*19	P467S
			CYP3A4*21	Y319C
			CYP3A4*23	R162W
			CYP3A4*24	Q200H
			CYP3A4*28	L22V
			CYP3A4*29	F113I
			CYP3A4*31	H324Q
			CYP3A4*32	I335T
			CYP3A4*33	A370S
			CYP3A4*34	I427V
			CYP3A4*35	L3V
	CYP3A5	Chloroquine, halofantrine, lumefantrine, arteether, artelinic acid	CYP3A5*8	R28C
			CYP3A5*9	A337T
